# Supplementary material for: SMCHD1 has separable roles in chromatin architecture and gene silencing that could be targeted in disease
Source: Nat Commun. 2023 Sep 25;14:5466. doi: 10.1038/s41467-023-40992-6 (PMC10519958; doi:10.1038/s41467-023-40992-6)
Supplement: Supplementary file 1 — Supplementary Information [file 41467_2023_40992_MOESM1_ESM.pdf]

Supplementary Figures

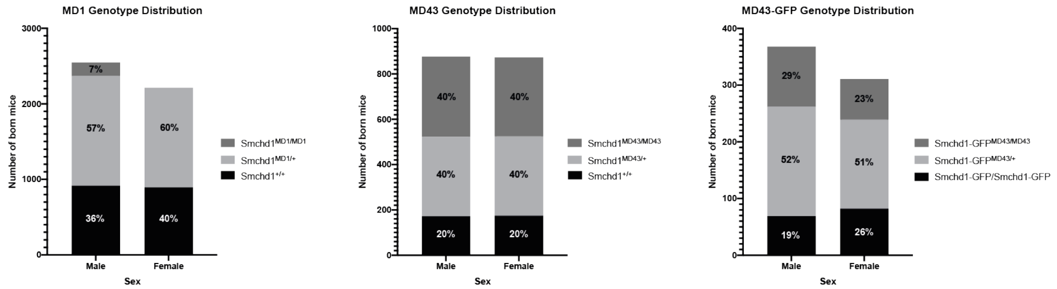

Total Offspring Born	5500
Total Offspring Weaned	4664 (84.80%)
Average Number of Offspring Born per Mating	7.199
Average Number of Offspring Weaned per Mating	6.701
Total Females Weaned	2174 (46.61%)
Total Males Weaned	2490 (53.39%)

Total Offspring Born	2155
Total Offspring Weaned	1837 (85.24%)
Average Number of Offspring Born per Mating	7.535
Average Number of Offspring Weaned per Mating	7.12
Total Females Weaned	916 (49.86%)
Total Males Weaned	921 (50.14%)

Total Offspring Born	1437
Total Offspring Weaned	836 (58.18%)
Average Number of Offspring Born per Mating	6.167
Average Number of Offspring Weaned per Mating	5.193
Total Females Weaned	378 (45.22%)
Total Males Weaned	458 (54.78%)

Supplementary Figure 1. Summarised genotype distribution data at weaning for offspring from for heterozygous intercrosses for *MommeD1*, *MommeD43* and *MommeD43-GFP*. *MommeD43* abbreviated to *MD43*. *MommeD1* abbreviated to *MD1*. Source data are provided as a Source Data file.

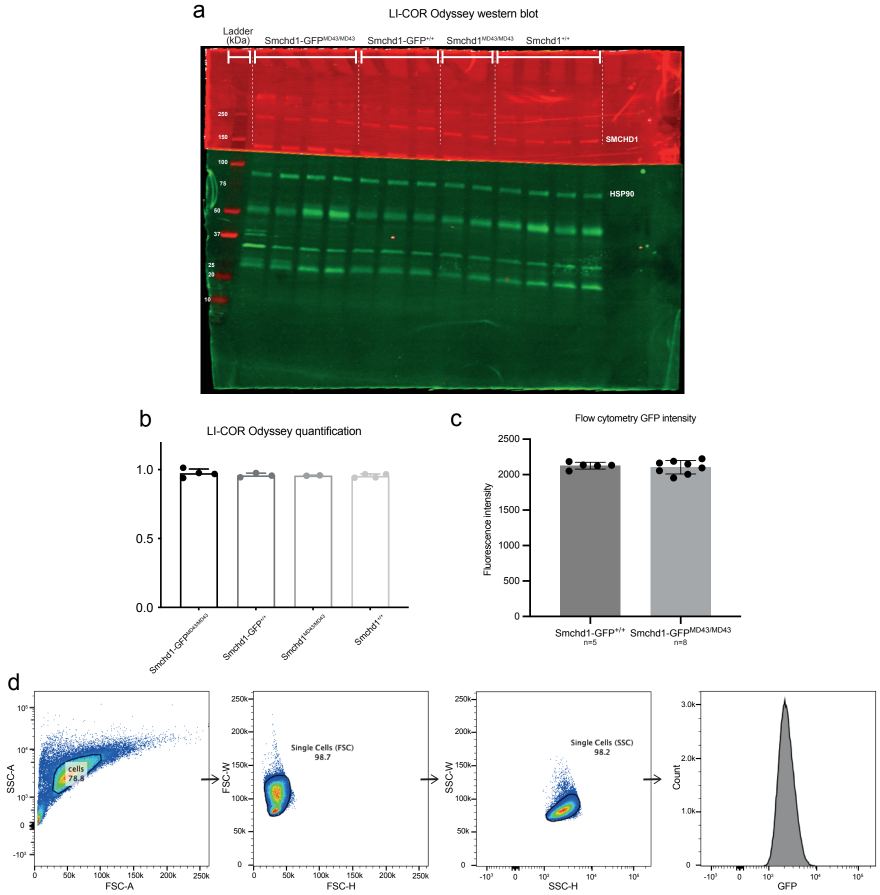

Supplementary Figure 2. a. LI-COR Odyssey quantitative western blot picture to measure levels of SMCHD1 protein in *Smchd1$^{GFP\text{-}MommeD43/GFP\text{-}MommeD43}$*, *Smchd1$^{GFP/GFP}$*, *Smchd1$^{MommeD43/MommeD43}$* and *Smchd1$^{+/+}$* primary MEFs (side-by-side lanes of the same genotype are biological replicates). b. Quantification (mean +/- SD) of the LI-COR Odyssey western blot shown in a. c. Median GFP fluorescence intensity in *Smchd1$^{GFP\text{-}MommeD43/GFP\text{-}MommeD43}$* and *Smchd1$^{GFP/GFP}$* primary MEFs measured by flow cytometry showing the same levels of SMCHD1 protein. Number of biological replicates (cell lines derived from different embryos) indicated below (mean +/- SD). d. Gating strategy for data shown in c. *MommeD43* abbreviated to *MD43*. Source data are provided as a Source Data file.

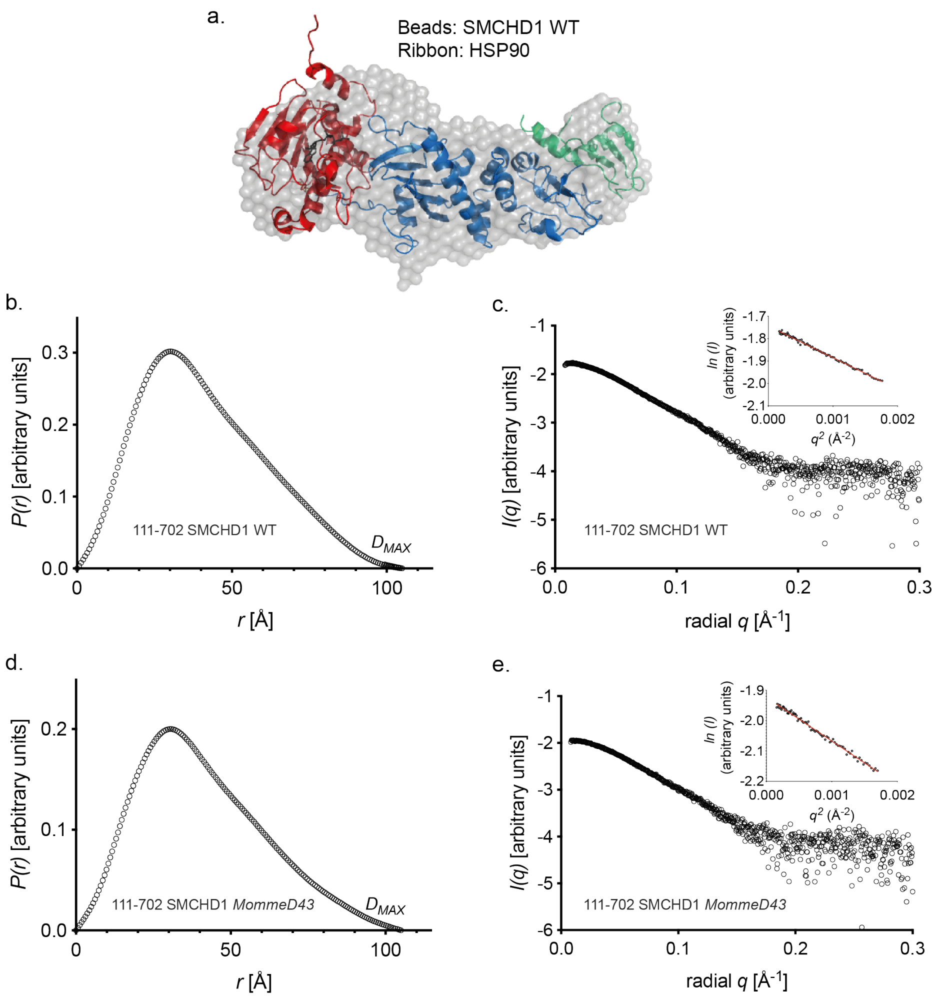

Supplementary Figure 3. a. Bead model of the extended ATPase domain (residues 111-702) of murine wild-type SMCHD1; data in b-e indicate SMCHD1 *MommeD43* has the same gross topology. Overlayed is the ribbon structure of the highly related GHKL ATPase domain of HSP90. b., d. Distance distribution function P(r) calculated from data shown in c. for wild-type SMCHD1 and e. for SMCHD1 MommeD43. c., e. Experimental scattering curves (black circles) of the recombinant ATPase domain (residues 111-702) of SMCHD1 WT and SMCHD1 MommeD43 respectively. On the right upper corner are the corresponding Guinier plots with the trend line from linear regression in red. Source data are provided as a Source Data file.

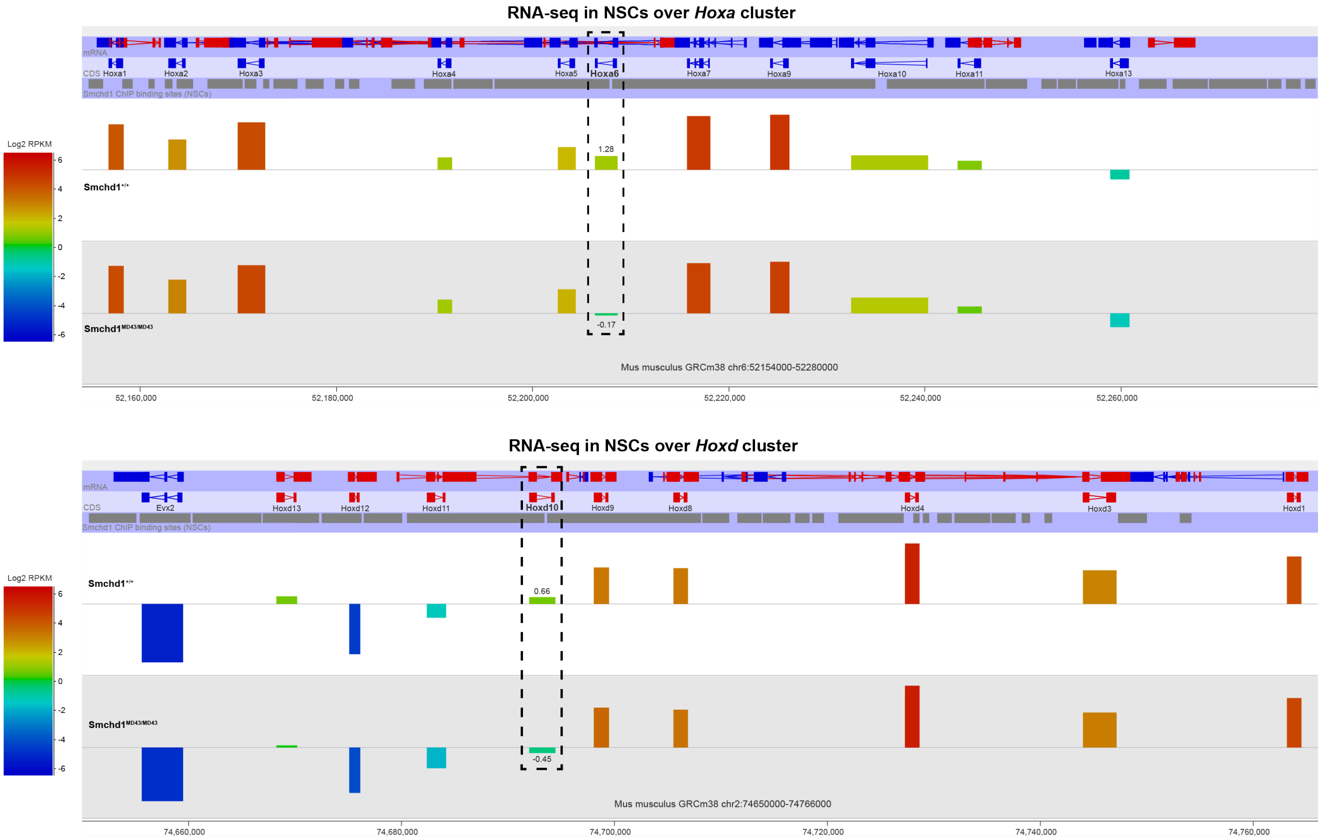

Supplementary Figure 4. Log2 RPKM counts over gene CDS in aggregated replicate sets of 8-somite pre-somitic mesoderm pools for *Smchd1*$^{+/+}$ and *Smchd1*$^{MD43/MD43}$ over the *Hoxa* cluster (top) and the *Hoxd* cluster (bottom). The grey track shows confirmed binding sites in NSCs over each cluster, and highlighted are *Hoxa6* and *Hoxd10* which show a significant difference in expression levels between both datasets.

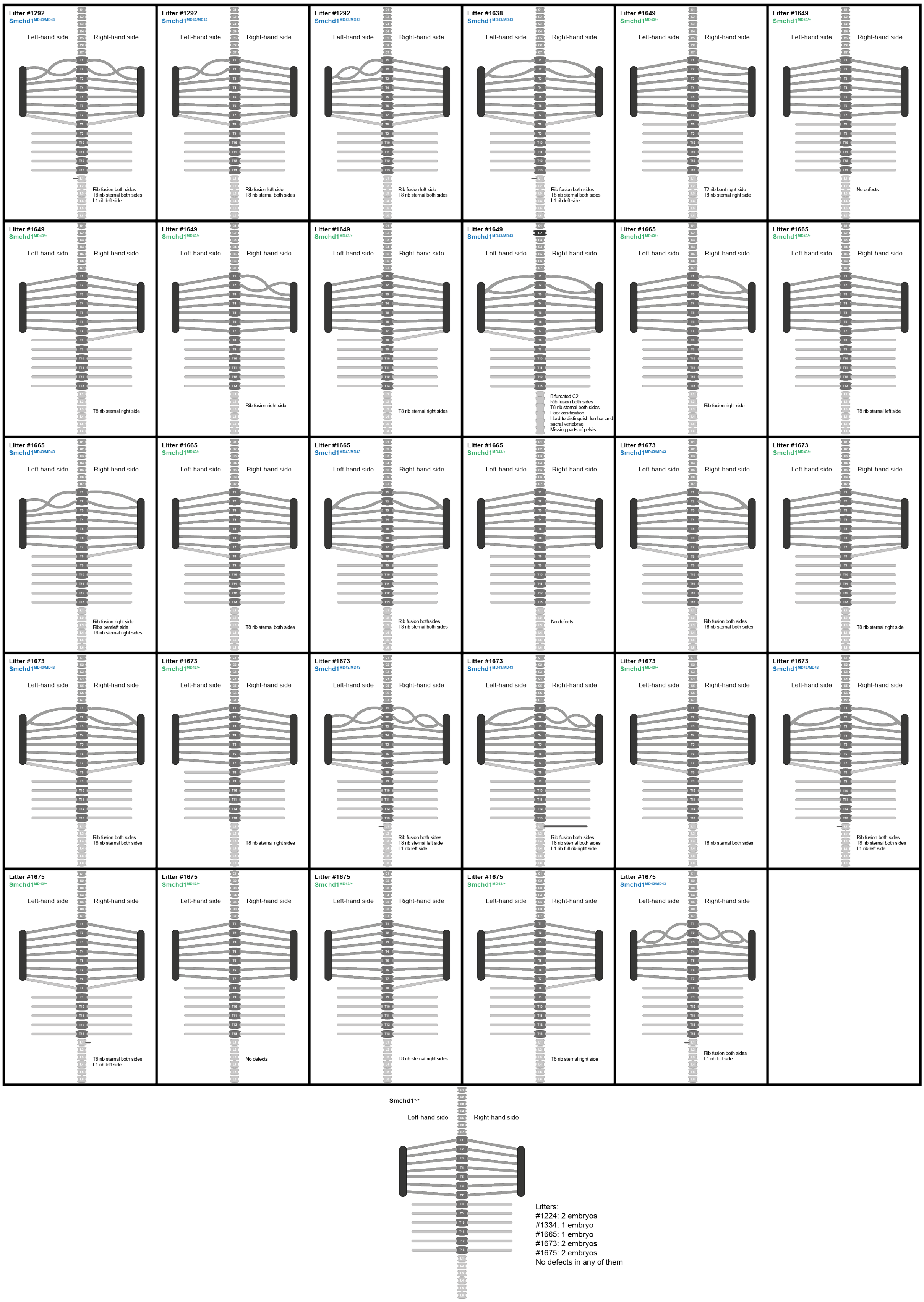

Supplementary Figure 5. Detailed scoring of skeletal defects observed in *Smchd1*$^{MommeD43/MommeD43}$, *Smchd1*$^{MommeD43/+}$ and *Smchd1*$^{+/+}$ E16.5 littermate embryos**.** Schematic representation of each embryo skeleton scored after skeletal preparation (37 in total). Only one diagram at the bottom represents all 8 Smchd1$^{+/+}$ embryos since all of them are identical. For the others, approximations of the shapes of the observed fused ribs were drawn. The view is dorsal and the sternum is represented on both sides by the dark vertical bar to which the T1 to T7 ribs join in the wild-type embryos. *MommeD43* abbreviated to *MD43*.

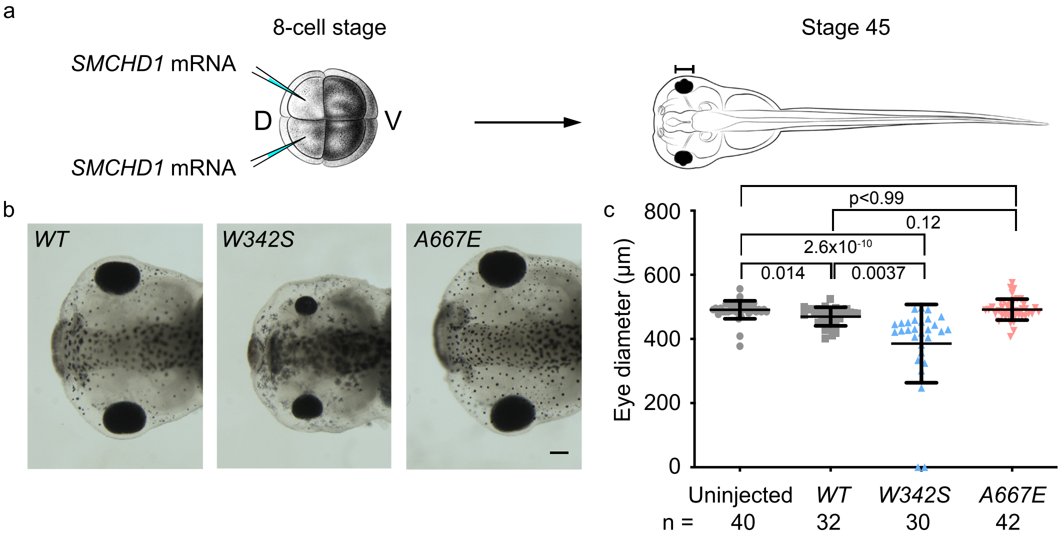

Supplementary Figure 6. Overexpression of *MommeD43* (A667E) mutation in *Xenopus laevis* embryos does not cause craniofacial anomalies. a. Schematic of experiment. *SMCHD1* mRNA was injected into the two dorsal (D) animal blastomeres at the 8-cell stage to target the future head of the tadpole. Eye diameters of the tadpoles were observed at Stage 45. b. Representative pictures of tadpoles injected with *SMCHD1* carrying various mutations. Scale bar represent 200 µm. c. Measurements of eye diameters in tadpoles injected with various SMCHD1 mRNA. W342S is a BAMS mutation, A667E is the *MommeD43* mutation. Results are given as means ± standard deviation, *p* values of each comparison under brackets (Kruskal-Wallis test with Dunn's correction for multiple comparison). Source data are provided as a Source Data file. Xenopus illustrations adapted from Xenbase and Zahn et al. (2022).
Xenbase (www.xenbase.org RRID:SCR_003280)
Zahn et al., Normal Table of *Xenopus* development: a new graphical resource. Development (2022) 149 (14): dev200356. https://doi.org/10.1242/dev.200356.

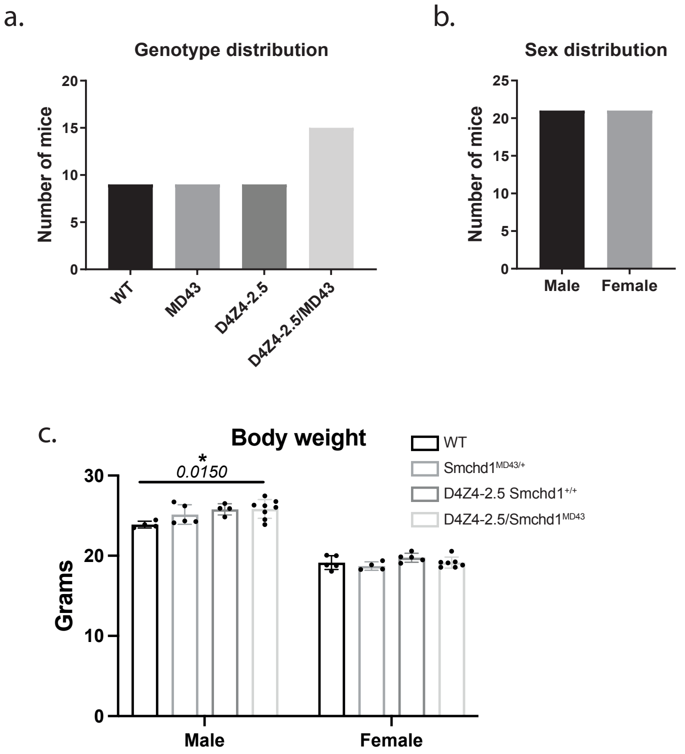

Supplementary Figure 7: a. Genotype distribution was not disturbed as tested by a Pearson's chi-squared test. b. An equal number of male (N=21) and female (N=21) mice were born. c. Body weight (in grams) of male and female mice at two months of age. Bars represent the average body weight per genotype; error bars denote the standard deviation. Statistical analysis was performed by two-way ANOVA followed by a

Sidak's test for post hoc analysis. WT = wild-type; MD43 = *Smchd1*$^{MommeD43/+}$. *P < 0.05, exact value indicated over bar. Source data are provided as a Source Data file.

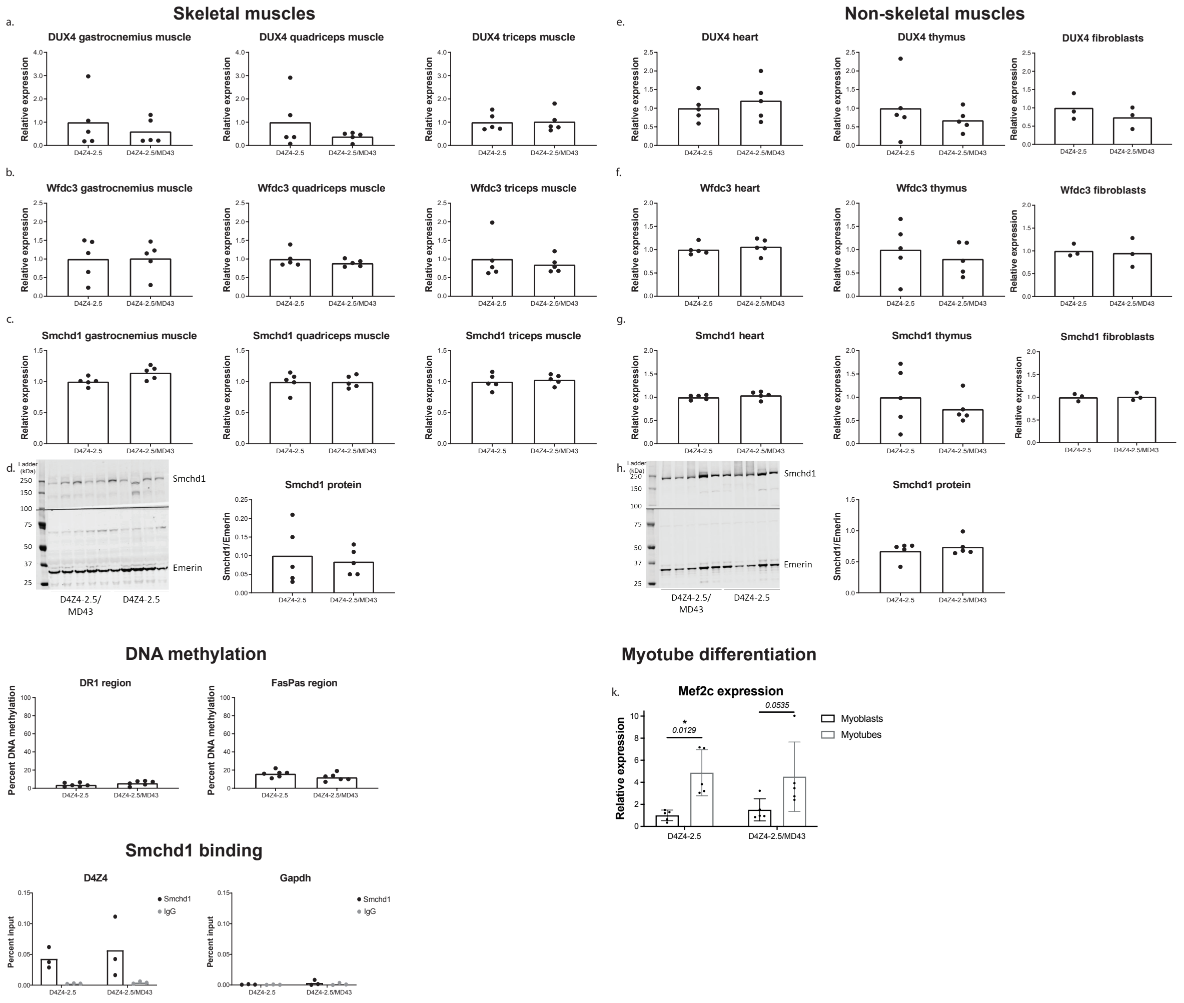

Supplementary Figure 8: a-c. Relative *DUX4* (a), *Wfdc3* (b), and *Smchd1* (c) transcript levels in three different skeletal muscles (gastrocnemius, quadriceps, triceps). d. SMCHD1 protein levels in tibialis anterior muscle. EMERIN was used as a loading control. e-g. Relative *DUX4* (e), f) *Wfdc3* (f), and *Smchd1* (g) transcript levels in heart, thymus and fibroblast cultures. h. SMCHD1 protein levels in spleen. EMERIN was used as a loading control. i. The average DNA methylation level of 19 CpG dinucleotides within the D4Z4 repeat (the DR1 region) and of 10 CpG dinucleotides just distal to the D4Z4 repeat (the FasPas region) in tail DNA. j. SMCHD1 binding at the *D4Z4* repeat and the *Gapdh* locus in fibroblast cultures. Bars represent the average DNA methylation levels or the SMCHD1 enrichment levels per genotype; each dot represents a single mouse. Bars represent the average transcript/protein levels per genotype (average value in D4Z4-2.5 group is set as 1 in a-c and e-g); each dot represents a single mouse (a-j). k. Relative *Mef2c* expression in myoblast and myotube cultures, two-tailed p values shown above (* denotes statistical significance). Bars represent the average transcript level per genotype (average value in D4Z4-2.5 myoblasts is set as 1); error bars denote the standard deviation. Statistical analysis was performed using a Student's t-test (a-j; except for the fibroblast cultures where a non-parametric Mann-Whitney U-test was used due to low sample number) or a two-way ANOVA followed by a Sidak's test for post hoc analysis. MD43 = *Smchd1*$^{MommeD43/+}$. Source data are provided as a Source Data file.

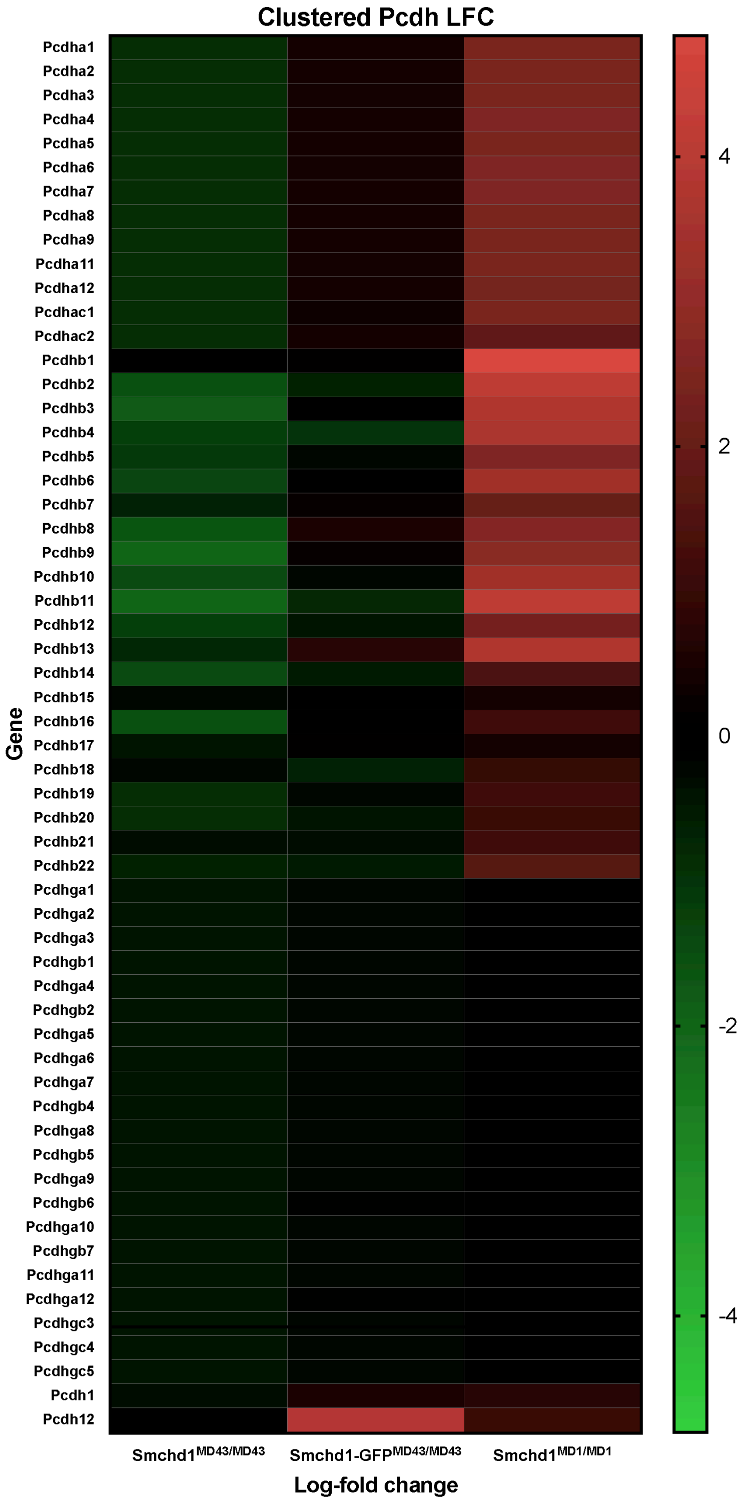

Supplementary Figure 9. Heatmap of log-fold change of clustered protocadherin genes in murine chromosome 18. The data is log2 normalized RPKM expression from mRNA sequencing data in *Smchd1*$^{MommeD43/MommeD43}$, *Smchd1*$^{MommeD43\text{-}GFP/MommeD43\text{-}GFP}$ and *Smchd1*$^{MommeD1/MommeD1}$ NSCs versus their independent controls *Smchd1*$^{+/+}$, *Smchd1*$^{GFP/GFP}$ and *Smchd1*$^{+/+}$, respectively. *MommeD43* abbreviated to *MD43*.

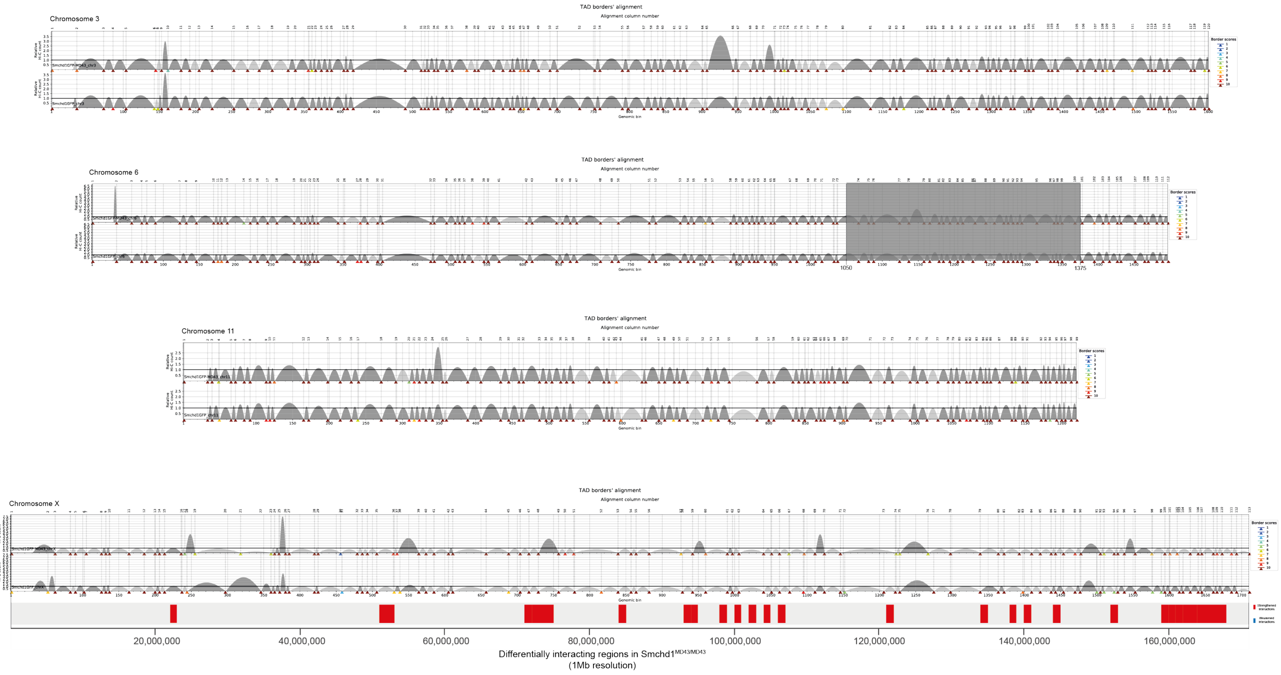

Supplementary Figure 10. TAD alignment diagrams from TADbit for chromosomes 3, 6, 11 and X at 100kb resolution. Each diagram shows an individual chromosome with TADs calculated from 3 merged biological replicates for *Smchd1-GFP*$^{MommeD43/GFP\text{-}MommeD43}$ top) and *Smchd1-GFP*$^{+/+}$ NSCs (bottom). TADs with relative Hi-C count >1 are in dark grey. The arrows on the X-axis represent TAD borders, their colour represents their statistical significance (border scores with 10 being highest significance). The Chr6:105Mb-137.5Mb region is greyed out as it was excluded from all analyses because of a chromosomal rearrangement present in the Smchd1-GFP$^{MD43/MD43}$ mouse colony. The units of the X-axis are genomic bins equal to the resolution (100kb). In addition, below the X chromosome's TAD alignment there is a track showing the statistically significant differentially interacting regions found in HiC data at 1Mb resolution in Smchd1-GFP$^{MD43/MD43}$ compared to Smchd1-GFP$^{+/+}$ NSCs (31 differential interactions FDR<0.1, all strengthened in *Smchd1-GFP*$^{MommeD43\text{-}MommeD43}$). *MommeD43* abbreviated to *MD43*.

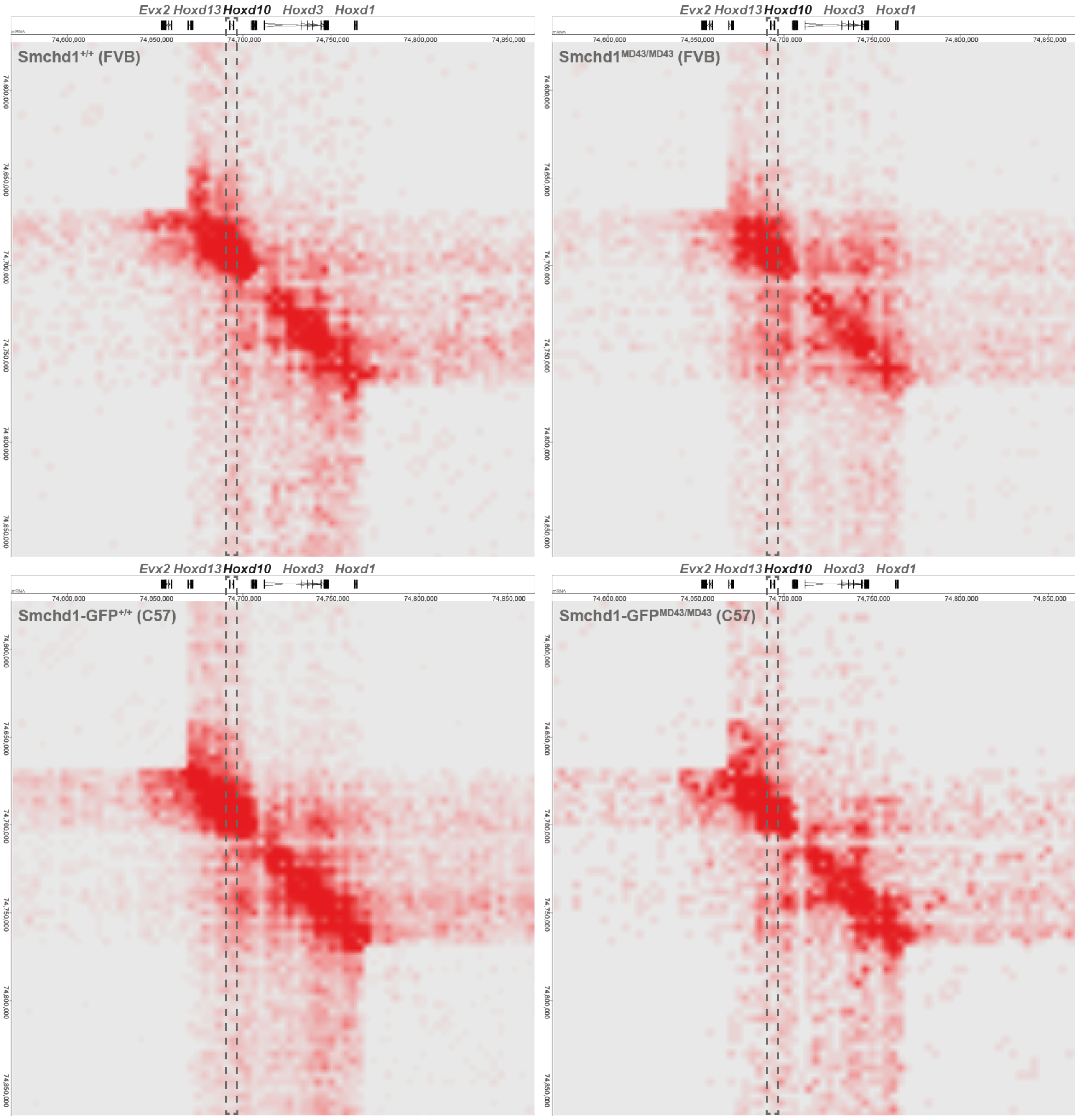

Supplementary Figure 11. Heatmaps of Capture-C interactions centered on the *Hoxd* cluster (*mm10* chr2:74668309-74765142) in pre-somitic mesoderm tissue from somite-matched embryos (E8.5). On top of each heatmap, a genome browser track shows some of the genes in the region. The dotted line encompasses interactions involving the *Hoxd10* locus, which showed altered expression in this tissue.

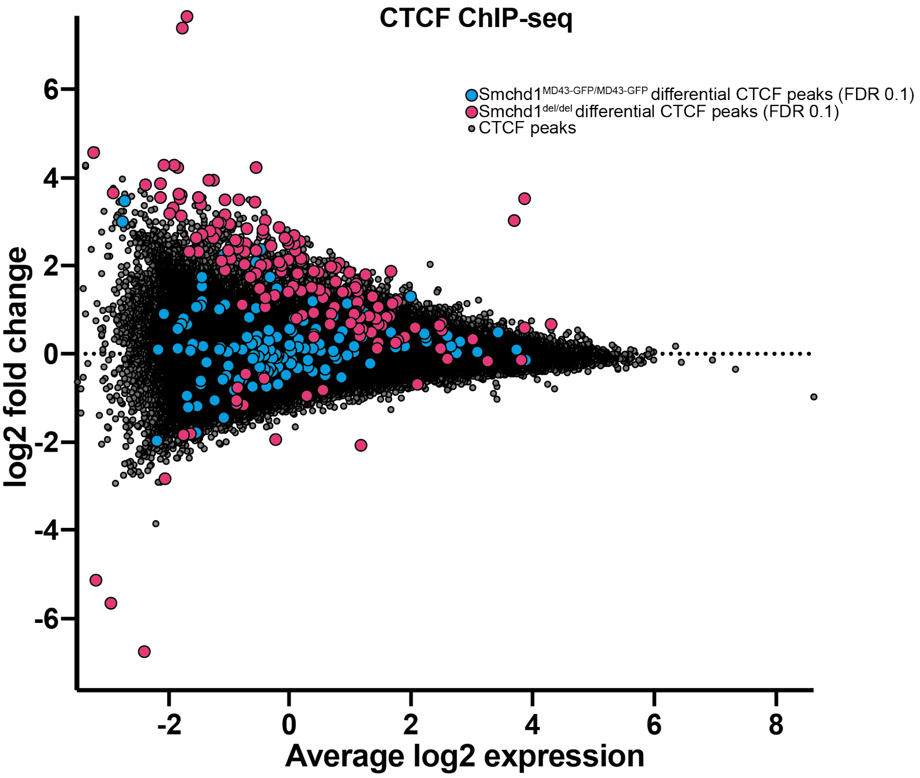

Supplementary Figure 12. MA plot showing normalized CTCF ChIP-seq values over peaks. On the x-axis, *Smchd1*$^{del/del}$ - *Smchd1*$^{GFP/GFP}$ log2 fold-change, and on the y-axis the average between them. Highlighted are peaks showing statistically significant differential binding, in cyan for *Smchd1*$^{MD43\text{-}GFP/MD43\text{-}GFP}$, and in magenta those found in *Smchd1*$^{del/del}$, both compared to *Smchd1*$^{GFP/GFP}$ (FDR 0.1).

Supplementary Tables:

Supplementary table 1

List of experiments with genotypes in which they have been replicated

Experiment	Genotypes studied	Tissue/cell type	Comments
Mouse genotype distribution data	*Smchd1*$^{+/+}$ (FVB/NJ)<br>*Smchd1*$^{MD43/MD43}$ (FVB/NJ)<br>*Smchd1*$^{GFP/GFP}$ (C57BL6/J)<br>*Smchd1*$^{MD43\text{-}GFP/MD43\text{-}GFP}$ (C57BL6/J)	Mice	
LI-COR Odyssey Western Blots for SMCHD1 protein levels	*Smchd1*$^{+/+}$ (FVB/NJ)<br>*Smchd1*$^{MD43/MD43}$ (FVB/NJ)<br>*Smchd1*$^{GFP/GFP}$ (C57BL6/J)<br>*Smchd1*$^{MD43\text{-}GFP/MD43\text{-}GFP}$ (C57BL6/J)	Neural Stem Cells (NSCs)	
Smchd1 ChIP-seq	*Smchd1*$^{GFP/GFP}$ (C57BL6/J)<br>*Smchd1*$^{MD43\text{-}GFP/MD43\text{-}GFP}$ (C57BL6/J)	Female NSCs	Used anti-GFP antibody. Not feasible in FVB/NJ relevant genotypes as there was no available anti-SMCHD1 antibody that worked for ChIP at the time.

ATPase assay	Recombinant extended ATPase domain of Smchd1	Purified recombinant protein	
SAXS	Recombinant extended ATPase domain of Smchd1	Purified recombinant protein	
Skeletal preparations for morphology study	*Smchd1*$^{+/+}$ (FVB/NJ)<br>*Smchd1*$^{MD43/MD43}$ (FVB/NJ)	Mouse E17.5 embryos	C57BL6J wild-type mice have frequent skeletal defects, therefore a very large number of embryos would be required to separate *MommeD43*-specific phenotypes in mice of that background.
RNA-seq in pre-somitic mesoderm (PSM)	*Smchd1*$^{+/+}$ (FVB/NJ)<br>*Smchd1*$^{MD43/MD43}$ (FVB/NJ)	Tailbuds containing PSM from somite-matched E8.5 embryos	Matched to the background used for skeletal preps. Embryology is easier in more robust FVB/N background lines which have larger litters.
HREM craniofacial morphology	*Smchd1*$^{+/+}$ (FVB/NJ)<br>*Smchd1*$^{MD43/+}$ (FVB/NJ)<br>*Smchd1*$^{MD43/MD43}$ (FVB/NJ)	Mouse E14.5 embryos	
Smchd1 overexpression in *Xenopus*	*Xenopus laevis*	*Xenopus* embryos<br>mRNA injected was human SMCHD1 with introduced mutations	
RNA-seq in frontonasal prominence (FNP)	*Smchd1*$^{+/+}$ (FVB/NJ)<br>*Smchd1*$^{MD43/MD43}$ (FVB/NJ)	FNP from somite-matched E10.5 embryos at the 29 somite stage	
Effects on DUX4 expression	D4Z4-2.5/*Smchd1*$^{+/+}$<br>D4Z4-2.5/*Smchd1*$^{MD43/+}$	Mice<br>Gastrocnemius, quadriceps and triceps<br>Cultured EDL muscle cells (myoblasts and differentiating myotubes)<br>Cerebellum, heart, spleen, thymus, fibroblast cell cultures	F1 cross C57BL6/J x FVB/NJ mouse strain, MommeD43 allele from the FVB/NJ line.
RNA-seq in NSCs	*Smchd1*$^{+/+}$ (FVB/NJ)<br>*Smchd1*$^{MD43/MD43}$ (FVB/NJ)<br>*Smchd1*$^{GFP/GFP}$ (C57BL6/J)<br>*Smchd1*$^{MD43\text{-}GFP/MD43\text{-}GFP}$ (C57BL6/J)<br>*Smchd1*$^{+/+}$ (FVB/NJ)<br>*Smchd1*$^{MD1/MD1}$ (FVB/NJ)	NSCs	
in situ HiC in NSCs	*Smchd1*$^{GFP/GFP}$ (C57BL6/J)<br>*Smchd1*$^{MD43\text{-}GFP/MD43\text{-}GFP}$ (C57BL6/J)	Female NSCs	
DNA FISH	*Smchd1*$^{+/+}$ (FVB/NJ)<br>*Smchd1*$^{MD43/MD43}$ (FVB/NJ)<br>*Smchd1*$^{GFP/GFP}$ (C57BL6/J)<br>*Smchd1*$^{MD43\text{-}GFP/MD43\text{-}GFP}$ (C57BL6/J)<br>Smchd1$^{fl/fl}$ (FVB/NJ)<br>*Smchd1*$^{del/del}$ (FVB/NJ)	NSCs	Results shown in Figure 5.i. are aggregated between strain backgrounds since they showed the same distribution.
Capture-C in pre-somitic mesoderm (PSM) from somite-matched E8.5 embryos	*Smchd1*$^{+/+}$ (FVB/NJ)<br>*Smchd1*$^{MD43/MD43}$ (FVB/NJ)<br>*Smchd1*$^{GFP/GFP}$ (C57BL6/J) and<br>*Smchd1*$^{MD43\text{-}GFP/MD43\text{-}GFP}$ (C57BL6/J)	Tailbud containing PSM from somite-matched (7-9 somites range) E8.5 embryos	
RRBS in NSCs	*Smchd1*$^{+/+}$ (FVB/NJ)<br>*Smchd1*$^{MD43/MD43}$ (FVB/NJ)	Female NSCs	
H3K27me3 ChIP-seq	*Smchd1*$^{GFP/GFP}$ (C57BL6/J) and<br>*Smchd1*$^{MD43\text{-}GFP/MD43\text{-}GFP}$ (C57BL6/J)	Female NSCs	Matched to *in situ* HiC data.
CTCF ChIP-seq	*Smchd1*$^{GFP/GFP}$ (C57BL6/J),<br>*Smchd1*$^{MD43\text{-}GFP/MD43\text{-}GFP}$ (C57BL6/J) and<br>*Smchd1*$^{del/del}$ (C57BL6/J)	Female NSCs	
Immunofluorescence for H3K27me3 and SMCHD1	*Smchd1*$^{+/+}$ (FVB/NJ)<br>*Smchd1*$^{MD43/MD43}$ (FVB/NJ)<br>*Smchd1*$^{GFP/GFP}$ (C57BL6/J)<br>*Smchd1*$^{MD43\text{-}GFP/MD43\text{-}GFP}$ (C57BL6/J)<br>Smchd1$^{fl/fl}$ (FVB/NJ)<br>*Smchd*$^{del1/del}$ (FVB/NJ)	Female NSCs	
Positional cloning in conjunction with linkage analysis utilising SNP chip technology followed by whole exome sequencing	*Smchd1*$^{+/+}$ (FVB/NJ)<br>*Smchd1*$^{MD43/MD43}$ (FVB/NJ)	Mouse tissue samples for genotyping	
Nanopore sequencing	*Smchd1*$^{+/+}$ (FVB/NJ)<br>*Smchd1*$^{MD43/MD43}$ (FVB/NJ)	Olfactory bulb from adult littermates	Resequencing to verify no off-target mutations in linkage region

			from ENU generation of MommeD43.

Supplementary table 2

Data collection and scattering parameters for SAXS analysis

**Data collection parameters**		
Instrument	Australian Synchrotron SAXS/WAXS beamline	
Beam geometry	120 µm point source	
Beam wavelength (Å)	1.033	
q range ($Å^{-1}$)	0.0114-0.4	
Exposure time (seconds)	2	
Protein concentration	~5 mg/ml sample injected via in-line size exclusion chromatography	
Temperature (°C)	16	
**Structural parameters**		
Protein sample	SMCHD1 111-702 WT	SMCHD1 111-702 A667E
I(0)(cm^{-1}) [from Guinier]	0.01806 ±0.00013	0.01196 ±0.00012
Rg (Å) [from Guinier]	31.2 ±0.344	31.7 ±0.484
I(0)(cm^{-1}) [from *P(r)*]	0.01825 ±0.00009	0.01198 ±0.00008
Rg (Å) [from *P(r)*]	32.17 ±0.206	32.21 ±0.273
D_{max} (Å)	105	105
**Software**		
Primary data reduction	ScatterBrain (Australia Synchrotron)	
Data processing	PRIMUS, GNOM	

Supplementary Table 3. Oligonucleotide sequences

Oligo name	Purpose	Sequence
D4Z4 gen F	D4Z4 transgene genotyping	5'- TGGTCCGTGAAGACATGTGT-3'
D4Z4 gen R	D4Z4 transgene genotyping	5'- GAGCCCTCAGAGAAGTGGG-3'
Smchd1 ex 15F	MommeD43 genotyping	5'-TTGTCTTATTTGCAATGTTGGTG-3'
Smchd1 ex 15R	MommeD43 genotyping	5'-GCTACAGTGCCTAGCCCAAT-3'
MommeD43 probe	MommeD43 genotyping	5'-AACTGTGCCCATTGCAAAGCTGGATAGGA-3'
D4Z4 ChIP F	D4Z4 ChIP-qPCR	5'- CCGCGTCCGTCCGTGAAA-3

D4Z4 ChIP R	D4Z4 ChIP-qPCR	5'-TCCGTCGCCGTCCTCGTC-3'
Gapdh ChIP F	Gapdh ChIP-qPCR	5'-TGAGCCTCCTCCAATTCAAC-3'
Gapdh ChIP R	Gapdh ChIP-qPCR	5'-CCAGGAAGACGCTTGAAAAG-3'
DR1 meth F	D4Z4 DR1 methylation	5'-GGGTTGAGGGTTGGGTTTATA-3'
DR1 meth R	D4Z4 DR1 methylation	5'- ACAAAACTCAACCTAAAAATATAC -3'
Faspas meth F	D4Z4 Faspas methylation	5'-ATAGGGAGGGGGTATTTTA-3'
Faspas meth R	D4Z4 Faspas methylation	5'-ACRATCAAAAACATACCTCTATCTA-3'
Otc F	X chromosome genotyping	5'-GTTCTTTCGTTTTCCCCTCTC-3'
Otc R	X chromosome genotyping	5'-GGCATTATCTAAGGAGAAGCATC-3'
Zfy F	Y chromosome genotyping	5'-GACTAGACATGTCTTAACATCTGTCC-3'
Zfy R	Y chromosome genotyping	5'-CCTATTGCATGGACAGCAGCTTATG-3'
Smchd1 cKO F2	Smchd1 fl and del genotyping	5'-TCAGGTGGTCTCGAGCCC-3'
Smchd1 cKO F4	Smchd1 fl and del genotyping	5'-CCATGAGAAGCAATGTGGGA-3'
Smchd1 cKO R1	Smchd1 fl and del genotyping	5'-GGACAGCCAAAGTGACACAG-3'
Smchd1 GFP F1	Smchd1-GFP genotyping	5'-GCCTGCCTTGCTTCTATGTC-3'
Smchd1 GFP R1	Smchd1-GFP genotyping	5'-GCCCCATGAGATTCTGAAAG-3'
Smchd1 GFP R2	Smchd1-GFP genotyping	5'-GAATTCAGGGTCAGCTTGC-3'
Momme D1 F	Allelic discrimination for genotyping of Smchd1	5'-TCCTCCTTGTGGCCTTGTGG-3'
MommeD1 R	Allelic discrimination for genotyping of Smchd1	5'-CGTATTTAAAGGTCCAGCTGTTGC-3'
Taqman Smchd1 wild-type probe used with MommeD1	Allelic discrimination for genotyping of Smchd1	VIC-CAGCTTTGGTTGTGCTGT-MGBNFQ
Taqman Smchd1 MommeD1 probe	Allelic discrimination for genotyping of Smchd1	6FAM-CAGCTTTGGTTATGCTGT-MGBNFQ
Taqman Smchd1 wild-type used with MommeD43 probe	Allelic discrimination for genotyping of Smchd1	VIC-TCCTATCCAGCTTTGCAAT-MGBNFQ
Taqman Smchd1 MommeD43 probe	Allelic discrimination for genotyping of Smchd1	6FAM-CCTATCCAGCTTTTCAAT-MGBNFQ
MommeD43 F	Allelic discrimination for genotyping of Smchd1	5'-CCCCAGGCACTGTATGATGAAATAA-3'

MommeD43 R	Allelic discrimination for genotyping of Smchd1	5’-CATATTTCCTAATTGTTTTCTCGGCTACTG-3’
sgRNA MommeD43	gRNA for CRISPR/Cas9	5’-TTGCAAAGCTGGATAGGACA-3’
Smchd1 exon 15 MommeD43	Oligo donor repair template for introducing the MommeD43 mutation by CRISPR/Cas9	5’-tgtaaatttttattcagGAACCCCAGGCACTGTATGATGAAATAAAAACTGTGCCTATTGAAAAGCTGGATAGGACAGTAGCCGAGAAAACAATTAGGAAATATGTAGAAGATGAAATGGC-3’
