# Supplementary material for: SMCHD1 has separable roles in chromatin architecture and gene silencing that could be targeted in disease
Source: Nat Commun. 2023 Sep 25;14:5466. doi: 10.1038/s41467-023-40992-6 (PMC10519958; doi:10.1038/s41467-023-40992-6)
Supplement: Supplementary file 3 — Description of Additional Supplementary Files [file 41467_2023_40992_MOESM3_ESM.pdf]

Description of Additional Supplementary Files

File Name: Supplementary Data 1
Description: List of single-nucleotide variants found in the linked interval in Smchd1MommeD43/MommeD43 mice from the ENU mutagenesis-generated colony with whole genome Nanopore long read sequencing.

File Name: Supplementary Data 2
Description: Normalized log2 RPKM counts for individual replicates of RNA-seq in tailbud tissue dissected at the 8 somite stage of embryonic development. Mean columns represent the average RPKM counts in either replicate set. WT = wild-type, MD43 = Smchd1MommeD43/MommeD43, LFC = log2 fold-change.

File Name: Supplementary Data 3
Description: Normalized log2 RPKM counts of differentially expressed genes (EdgeR, p<0.05) in 29 somite frontonasal prominence tissue dissected from E10.5 embryos (Smchd1MommeD43/MommeD43 compared to Smchd1+/+). The merged replicates columns contain the average log2 RPKM counts from both libraries in each replicate set. WT = wild-type, MD43 = Smchd1MommeD43/MommeD43, FDR = false discovery rate.

File Name: Supplementary Data 4
Description: Normalized log2 RPKM counts of differentially expressed genes (EdgeR, p<0.05) in 29 somite frontonasal prominence tissue dissected from E10.5 embryos (Smchd1MommeD1/MommeD1 compared to Smchd1+/+). The merged replicates columns (named 'reps') contain the average log2 RPKM counts from all libraries in each replicate set. WT = wild-type, MD1 = Smchd1MommeD1/MommeD1, FDR = false discovery rate.

File Name: Supplementary Data 5
Description: Normalized log2 CPM HiC interaction counts in Smchd1MommeD43-GFP/MommeD43-GFP NSCs (aggregated replicates) which were found to be differentially interacting (diffHiC, FDR<0.1) in Smchd1MommeD43-GFP/MommeD43-GFP versus Smchd1GFP/GFP at 1Mb and 100kb resolution, and normalized log2 CPM HiC interaction counts in Smchd1del/del NSCs which were found to be differentially interacting in Smchd1del/del versus Smchd1flox/flox at 1Mb and 100kb resolution. MD43 = Smchd1MommeD43/MommeD43, Smchd1 del = Smchd1del/del, logFC = log2 fold-change between corresponding Smchd1 variant and its control, FDR = false discovery rate.

File Name: Supplementary Data 6
Description: Normalized counts of detected significant interactions (CHiCANE, q value<0.05) in Capture-C data in tailbud tissue from 7-9 somite embryos. Columns 'count.1-4' contain the values for each individual replicates, while 'count' contains their aggregate. GFP_GFP = Smchd1GFP/GFP, MD43-GFP_MD43-GFP = Smchd1MommeD43-GFP/MommeD43-GFP, +_+ = Smchd1+/+, MD43_MD43 = Smchd1MommeD43/MommeD43, q.value = p value corrected for multiple testing.

File Name: Supplementary Data 7
Description: Normalized counts from H3K27me3 ChIP-seq data over peaks detected in Smchd1MommeD43/MommeD43 and Smchd1+/+ neural stem cells. Columns 'rep1-3' contain the counts for each individual replicate, and 'merged_reps' contain the average within the replicate set. MommeD43 = Smchd1MommeD43/MommeD43, WT = wild-type, WCE = whole cell extract (input).

File Name: Supplementary Data 8
Description: Log2 normalized counts from aggregate replicates in ChIP-seq for CTCF in Smchd1GFP-MommeD43/GFP-MommeD43, Smchd1del/del and Smchd1+/+ female NSCS over all peaks detected in either test-control comparison. The column 'Differential binding' indicates if the peak was found to be differentially bound (CSAW, FDR<0.1) in each comparison. WT = Smchd1GFP/GFP, MD43 = Smchd1MommeD43-GFP/MommeD43-GFP, DEL = Smchd1del/del.

File Name: Supplementary Data 9
Description: Illumina GoldenGate genotyping assay (Mouse Medium Density Linkage Panel) data from 11 phenotypically mutant and 11 phenotypically wild-type littermates. LOD = logarithm of the odds.
