# Supplementary figures and images for: SMCHD1 has separable roles in chromatin architecture and gene silencing that could be targeted in disease
Source: Nat Commun. 2023 Sep 25;14:5466. doi: 10.1038/s41467-023-40992-6 (PMC10519958; doi:10.1038/s41467-023-40992-6)

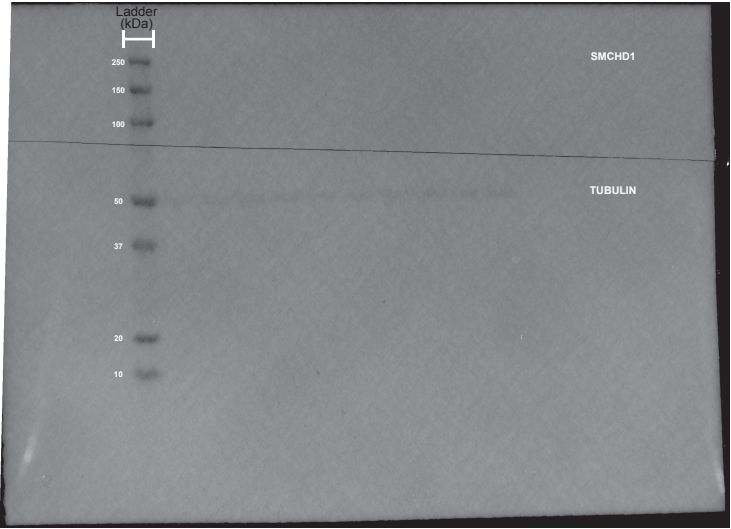

Supplement: Supplementary file 14 — Source Data [file 41467_2023_40992_MOESM14_ESM.zip › Source_Data/Figure_1c_uncropped_Western_Blot.pdf]
